# A new conceptual framework for investigating complex genetic disease

**DOI:** 10.3389/fgene.2015.00327

**Published:** 2015-11-04

**Authors:** Shobbir Hussain

**Affiliations:** Department of Biology and Biochemistry, University of BathBath, UK

**Keywords:** somatic mosaicism, mutator alleles, anti-mutator alleles, nanopore sequencing, rare variants, RVAS, genome wide association studies, rare variant association studies

## Abstract

Some common diseases are known to have an inherited component, however, their population- and familial-incidence patterns do not conform to any known monogenic Mendelian pattern of inheritance and instead they are currently much better explained if an underlying polygenic architecture is posited. Studies that have attempted to identify the causative genetic factors have been designed on this polygenic framework, but so far the yield has been largely unsatisfactory. Based on accumulating recent observations concerning the roles of somatic mosaicism in disease, in this article a second framework which posits a single gene-two hit model which can be modulated by a mutator/anti-mutator genetic background is suggested. I discuss whether such a model can be considered a viable alternative based on current knowledge, its advantages over the current polygenic framework, and describe practical routes via which the new framework can be investigated.

## Introduction

Some diseases within the human population are described as being ‘complex’ or ‘multifactorial’ in that they have an underlying inherited genetic etiology which contributes to disease risk along with environmental factors. These occur relatively common within the population and examples include congenital disorders such as cleft lip and palate, pyloric stenosis, clubfoot, and neural tube defects; those that can manifest at some point in childhood/early adulthood such as autism and schizophrenia; and those that can appear later in life such as Alzheimer’s disease. The proportion of genetic and environmental contributions to these types of diseases varies, but the proportion of variance of each of these particular diseases observed within a population which is due to variance in genetic factors is sometimes referred to as the ‘heritability’ and can be calculated via multiple methods. The simplest method, to obtain an estimate, is by observing the difference in concordance for the disease between monozygotic and dizygotic twins. The aforementioned diseases as well as several others are estimated to have a high heritability. The current consensus is that this genetic contribution is polygenic, such that multiple genetic loci interact in some fashion in order for the disease to manifest and such a model was put forth primarily based on the observed patterns of population- and familial-incidences of such diseases. In the last decade or so, various population-based large scale efforts to unmask the precise genetic factors responsible for disease manifestation have been based on this model, and have included investigations probing genetic variations that occur commonly, and more recently those that occur more rarely within the population. Overall, the output from such investigations have been disappointing in terms of providing little in the way of identifying and characterizing causal variants that actually play a significant role in disease manifestation. Here, I propose and describe an alternative theoretical model and consider whether the new model would be consistent with the population and manifestation characteristics of the diseases as well as current knowledge in genetics. I also discuss the merits and potential flaws of such a framework in comparison to current models, and define routes by which the framework could be tested and utilized.

## Polygenic Theory, Disease-Threshold, And The Carter Effect

Fisher (1918), arguably one of the most important thinkers in the history of the biological sciences, published a work which he entitled ‘The correlation between relatives on the supposition of Mendelian inheritance’. It was a landmark moment in our understanding of the genetics of inheritance. With the use of several mathematical applications, which many geneticists would struggle to grasp the inner-workings of, he settled an ongoing and at times heated debate between two major schools of thought regarding fundamental aspects of inheritance. On one side were the ‘Mendelians’ who championed the ideas of Mendel that single entities, now known as genes, were sufficient to determine phenotypic traits; and on the other side the statistically inclined ‘biometricians’ who argued that the majority of phenotypic traits displayed a continuous variation within the population and therefore at odds with this Mendelian idea of inheritance which rather predicted a dichotomous appearance of characters. Fisher’s (1918) mathematical intuition and training enabled him to introduce novel statistical models to demonstrate that ‘cumulative Mendelian factors seem to fit the facts very accurately’ as he put it; meaning that several genetic factors contribute in an additive manner to determine the value of an observed continuous trait. This came to be known as ‘polygenic theory’ and the work further laid the foundations of ‘quantitative genetics’ which concerns the study of genotype–phenotype correlations within populations.

The statistical models, Fisher (1918) introduced in his seminal paper and in further works (Fisher, 1922) are still widely used today in various fields of science. Though Fisher (1918) developed polygenic theory with the particular intention to explain continuous traits within populations, shortly following his death, his theory was extended in an attempt to explain the presence of dichotomous characters (Falconer, 1965), i.e., those characters previously acknowledged to be consistent with a Mendelian single-gene model. There was good motivation behind making such an extension: it was observed that many common diseases of a dichotomous nature that displayed some degree of familial clustering, displayed a rather low recurrence incidence amongst relatives and thus did not conform to any known pattern of Mendelian inheritance. Already inherent to Fisher’s (1918) polygenic model was that an individual is much less likely to develop the disease if he/she needs to inherit several alleles in order for the disease to manifest compared to if he/she needed to inherit just one or two (as in Mendelian inheritance), thus potentially explaining the reduced familial recurrence risks. However, to reconcile the observations of the dichotomous nature of diseases with Fisher’s (1918) model, the idea of ‘threshold’ was included where it is postulated that many genes do in fact contribute to disease manifestation in an additive fashion, but that only when a particular threshold biological value is breached, does the disease manifest. Thus the ‘polygenic threshold model’ as it became known neatly ticked all the required boxes, and in one form or another, has remained a mainstay as an explanation for inherited diseases which do not conform to any classic Mendelian pattern whilst displaying a dichotomous manifestation.

Indeed, this very concept of disease-threshold was incorporated into an explanation for another more specific observation regarding the population characteristics of some of these diseases: that a few displayed sex dimorphism where one particular sex is more commonly affected than the other (Carter and Evans, 1969). For example, in some disorders males are more commonly affected, and it is proposed that females must possess a higher threshold for disease expression: therefore a higher number of disease susceptibility alleles need to be inherited for the disorder to manifest in females. As a consequence, this would also mean that when females are affected, it would suggest a higher ‘genetic load’ of disease susceptibility alleles within the family and thus a higher recurrence risk for her relatives. Thus in a sex dimorphic polygenic disease, when the usually less commonly affected sex presents with the disease within a family, there is predicted to be a higher recurrence risk for relatives. This phenomenon became known as the ‘Carter effect’.

It may be of some note at this point that Fisher (1918) appeared quite happy to let the Mendelians ‘have’ the dichotomous traits, as is quite apparent from the fact that he made little effort to explain them using his polygenic theory, though he had plenty of scope, and no doubt, the intellectual vision to do so. Further, it is imperative to point out here that though the later proposed disease-threshold model can sometimes be useful in helping to estimate the recurrence risk of a disease once the population- and familial-incidences are known, as well as in making heritability predictions, there is currently no direct evidence to support the model. Indeed, it should at least be of a slight concern that the Carter effect is widely cited as evidence for the threshold model, as conversely there is no evidence to support the basis of the Carter effect, nor even a proposed biological mechanism via which one particular sex would have a higher/lower threshold for the expression of diseases.

## From Common Variants To Rare Variants

Rarer inherited diseases tend to display a monogenic Mendelian pattern of inheritance within families, and toward the end of the last century, linkage and mapping studies had been very successful in identifying the genetic causes of many of these. However, similar types of studies proved insufficient for identifying the causes of more common inherited diseases suspected to have an underlying polygenic determination. Indeed this difficulty was attributed to the existence of several causative genes, each only contributing a small-effect on disease manifestation, making them difficult to pinpoint using standard genetics approaches. Human genome sequencing projects earlier on this century, however, revealed that some genetic variations occurred fairly commonly within the population, usually as single nucleotide polymorphisms (SNPs). It was hypothesized that these common genetic variants were likely to be responsible for polygenic disease manifestation, i.e., phenotypic variance would be dependent on the contribution of several genetic variants each of which would make only a small contribution toward the manifestation of the disease; and thus these genetic variants needed to occur fairly commonly within the population to account for the prevalence of the disorders. This became known as the ‘common disease-common variant’ hypothesis, and it seemed somewhat fitting that technological advances being made at the time meant that this could be tested using physical molecular genetics experiments in approaches termed genome wide association studies (GWAS). The highs and lows of GWAS studies have been discussed and debated extensively elsewhere and such a discussion is not necessary here. But to cut a long story short, the current consensus is that these common genetic variants at most account for very little of the causative genetic contribution to these diseases.

Focus in the field has more recently turned its attention toward rare genetic variants to search for this otherwise ‘missing heritability’ (Manolio et al., 2009). In this case it is hypothesized that low frequency genetic variants may have larger-effect sizes, thus with fewer alleles needing to contribute in a polygenic manner toward disease manifestation. Investigations to identify these causative rare genetic variants in patients can now be performed with next-generation-sequencing technologies and are sometimes referred to as Rare-Variant-Association-Studies (RVAS). Investigations have shown that rare genetic variants are highly abundant in human populations and indeed that these variants are more likely to have larger deleterious biological effects (Nelson et al., 2012; Tennessen et al., 2012) as predicted by the ‘rare-variant-common disease’ hypothesis. Indeed, it has been suggested that an extensive ‘polygenic burden’ of rare variants within individuals might contribute to the prevalence of the diseases within human populations (Purcell et al., 2014). Currently, the main consensus is that the case/control population cohort sizes would need to be substantially large for the actual causative variants to be identified, thus making efforts to identify disease-causing genes very challenging (Derkach et al., 2014; Lee et al., 2014; Robinson et al., 2014; Sham and Purcell, 2014; Zhang, 2015). There are additional concerns over the reliability of *in silico* methods that are being used to predict whether rare-variants are likely to be deleterious (Henn et al., 2015). Further, the RVAS studies that have been performed so far indicate that most rare variants in fact only have modest-to-small effect sizes on phenotypic variation (Auer and Lettre, 2015). However, it is still relatively early days for such RVAS studies, and as GWAS studies did until a few years ago, they currently dominate the complex genetic diseases research landscape.

## An Alternative Theoretical Framework

When quizzed by an astute undergraduate student, ‘why such a big fuss about rare variants now?’ it is somewhat embarrassing to have no much better answer than ‘because we were mostly wrong about common variants’. We could attempt to lessen the blow by explaining that we are technologically in a much better position today to investigate rare variants in the laboratory, but in truth the student has a point: since both hypotheses were proposed, little has changed regarding our overall understanding of these variants and nothing has changed regarding the theoretical frameworks upon which both of the hypotheses were synthesized. Thus without the hindsight that the common variant hypothesis is deficient, there is little that we have learnt to suggest that the polygenic rare variant hypothesis is any more scientifically sound (Gibson, 2012; Robinson et al., 2014; Auer and Lettre, 2015; Henn et al., 2015). This makes it very acceptable, even prudent, to seriously consider alternative theoretical frameworks, even though they may be against major consensus held for decades. Here, I attempt to piece together a consistent framework which is based on empirical and experimental observations of colleagues in the field, past and present. The fundamental aspects of the conceptual framework are outlined in **Box 1**; and the rationale, merits, and potential shortcomings, are discussed in the sections below.

**Box 1. HSS model predictions.**

(1) A generally high, but varying, degree of locus heterogeneity exists for these diseases.
(2) Biallelic inactivation of a single gene is both necessary and sufficient for disease manifestation.
(3) The first mutation is inherited from a carrier parent. A random second hit occurs somatically; most likely at some rapid growth phase during embryonic/fetal development, but may also occur during childhood.
(4) Genetic variants which are inherited from both parents alter the frequency of an individual’s mutation rate, and these are a component of the genetic background. Several such mutator/anti-mutator alleles contribute in an additive fashion to modulate the probability of the second hit occurring.
(5) When sex dimorphism is observed, it can be explained by X-linked inheritance within some families, though with deviations from the expected classic Mendelian pattern, rather than the Carter effect.
(6) The framework is more likely to apply to diseases with a dichotomous manifestation and to diseases with a suspected developmental component, but may not be limited to diseases of this character.
(7) Environmental factors might contribute to disease risk, but in a lot of cases, they play a much lesser role than predicted by the current multifactorial model.

## Considering The General Patterns Of Observed Familial- And Population-Incidences

### A Single-Gene Two-Hit Model

Much of the inspiration for writing this article came from considering observations and ideas of a study entitled ‘Genetics, Chance, and Morphogenesis’ by Kurnit et al. (1987) published almost 30 years ago. The study investigated the roles of natural random probabilistic events that occur during early development, such as endocardial cell migration, and their influence over biological process by performing computer simulations. They observed a high degree of probabilistic variability in the outcomes of their simulations, which was distributed continuously: and in fact, the output appeared to resemble the normal distribution curves predicted by polygenic theory. They further demonstrated that this also held true if they included a single gene into their probabilistic model, and propose that multifactorial diseases may thus in fact be caused by inheritance of one or two copies of a single defective gene but also by chance events that occur during normal embryonic development as an important contributing factor. A limitation acknowledged by Kurnit et al. (1987) and extended on later by colleagues (Hook, 1988), was that the model didn’t satisfactorily account for steep falling-off of recurrence risk with diminishing relatedness as well as it should (addressed in ‘Mutator/Anti-mutator Genetic Background section’ below). Further, whereas the occurrence of sporadic birth defects may be attributed to some random very bad luck event during development, whether such events occur frequently enough to interact with genetic factors to significantly contribute to relatively common diseases within the population is perhaps much less likely. Here, I suggest a variation to the model such that only one defective copy of a single gene is inherited; and that rather than an adverse random biological processes during development, the chance element comes from inactivation of the second copy of the gene via random mutation during early development or growth. This two-hit model would explain the reduced recurrence-risk within affected families compared to classic Mendelian disorders.

Such a two-hit model is of course at the foundation of our understanding of how mutations in tumor suppressor genes contribute to some forms of cancer (Knudson, 1971). Cancerous cells and tissue represent special examples where accumulating mutations in cell cycle regulators and DNA repair factors accelerate the rates of mutation such that likelihood of the second-hit is increased, and where a new mutations can confer a growth advantage to the cell, thus expanding the target for the next cancer-promoting mutation to occur. The primary reason why such a model is not normally extended to non-cancerous disease is that such events of successive growth-promoting mutations are unlikely in healthy tissue. However, the model presented here posits that only a single mutation needs to occur for disease manifestation, and that this most likely occurs during a rapid growth phase such as in embryonic or fetal development, where a rapid rate of cell division is already naturally inherent to most cells. It is also important to note here that most gene disruptions will not significantly alter growth rates, whether it’s a reduction or an increase, or trigger apoptosis, as is sometimes erroneously assumed. Depending on how early these mutations occur in development they can either effect an entire organ or physiological system, or specific circuits within an organ – but enough to manifest as disease. Highly complex organs, such as the brain, probably require a smaller amount of tissue to be affected for the disease to manifest. Such considerations would also explain why these diseases would not display a classic Mendelian pattern, i.e., when both parents are carriers: if both mutations are inherited this would result in all cells in the offspring being affected, immediately from the single-cell zygote stage, and thus a different observed phenotype. A rather striking example of this comes from our knowledge of mutations in the BRCA2 gene: when one mutation is inherited, there is a predisposition to breast cancer; whereas when two mutations are inherited it causes a congenital syndrome, Fanconi’s anemia, where patients instead display high incidences of leukemia, bone marrow failure, and other wide and varying phenotypes (Howlett et al., 2002).

What is a crucial consideration, however, is the natural mutation rate within healthy tissues. This is still an area of active enquiry and estimates vary (Callaway, 2015). But what is clear is that estimates of the somatic mutation rate are consistently considerably much higher than that in the germline (Garcia et al., 2007; Lynch et al., 2008; Lynch, 2010a,b). The appearance of mutations within our genomes include errors incorporated during replication as well those inflicted by mutagens, but an additional very important source is the commonly occurring spontaneous deamination of methylated cytosines of CG dinucleotides to thymine. CG dinucleotides have generally reduced in frequency in genomes over history due to these spontaneous deamination events, but they remain more frequently in exons of protein coding genes where their presence to encode the correct sequence of amino acids is more crucial. Consequently, when deaminations at these sites do occur, they are very often identified as hotspots of causative point mutations in Mendelian and sporadic genetic disease. No truly reliable estimate of somatic mutation rate, however, exists, and this is largely due to our inability over the years to perform a wide survey to reliably determine the spectrum of sequence changes within the genomes of multiple tissue regions, although it is noteworthy that important initiatory studies in this regard using modern sequencing and computational approaches find very extensive somatic genetic variation when diverse tissues are sampled (Frank, 2010; O’Huallachain et al., 2012; Behjati et al., 2014); and more common roles for somatic mosaicism in disease manifestation has been suggested (De, 2011; Cohen et al., 2015). Indeed, our observations of non-cancerous human diseases caused by somatic mutations has been steadily increasing in recent years as our ability to detect them improves; and this includes mutations that may accumulate during the lifetime of an individual in age-related illnesses (Forsberg et al., 2012, 2013; Bonnefond et al., 2013; Aghili et al., 2014), and also new mutations that occur in early development that may contribute to the formation of sporadic forms of non-cancerous disease (Lindhurst et al., 2011; Poduri et al., 2012, 2013; Li et al., 2013; Dal et al., 2014).

### A Mutator/Anti-mutator Genetic Background

Mutation rates vary between species and they can also be influenced by variant alleles of genes encoding DNA replication and repair machinery within populations (Fijalkowska and Schaaper, 1995; Wibley et al., 2003; Baer, 2007; Senejani et al., 2012). Further, the observation that germline and somatic mutation incidences are often widely different within eukaryotic organisms indicates the existence of factors, likely genetically encoded, that can exert control over the rate of mutation (Lynch, 2010b). In addition, there are likely several yet other diverse genetically encoded factors that can influence mutation rate by regulating the rates/efficiency of mutagen clearance within cells. Importantly, other classes of genetically encoded factors that can influence the mutation rate have also been described; and these are currently known to range from microRNAs, to factors which regulate dNTP pools, amongst others (Valeri et al., 2010a,b; Tili et al., 2011; Ahluwalia and Schaaper, 2013; Alderton, 2013; Scanlon and Glazer, 2015; Sohl et al., 2015). Thus we likely have an architecture where genetic variants of a very broad range of cellular factors capable of significantly influencing the mutation rate exist, thus potentially resulting in significant variance of the mutation rate amongst individuals within a population.

With regards to the two-hit model presented in the current framework, once the first mutation has been inherited, since the chance second hit is both necessary and sufficient for disease manifestation, any factors that influence the probability of the second hit occurring, will play a significant role in determining disease manifestation. We know that classic Mendelian disease can sometimes be influenced by the genetic background which can modulate the penetrance. Here, it is proposed that the genetic background also otherwise contains an additional very important component that contributes to disease manifestation in this particular framework: a number of mutator and anti-mutator alleles; and that these variants can be inherited from both the carrier-parent and the non-carrier parent. Such a proposal would help explain the steeper falling-off of recurrence risk with decreasing relatedness observed for these diseases compared to the proportionate falling-off observed in classic Mendelian disorders. This is perhaps the most important aspect of this entire framework, and may be indirectly supported by observations. Mutation rates are known to be elevated in multicellular species relative to unicellular organisms, and in a fascinating theoretical undertaking, Lynch (2008) hypothesizes that multicellularity increases the probability of fitness reduction associated with somatic damage, but paradoxically in addition actually encourages the accumulation of mutator alleles that cause such damage, thereby magnifying the vulnerability to somatic mutations and disease. He also notes that when mutator alleles rise to moderate frequencies, considerable heterogeneity in the mutation rate is expected among individuals. The general idea of genetic variants commonly influencing mutation rates across a population is supported by a large-scale sequencing study which revealed that mutation rates vary substantially between families, and the authors thus suggest that mutation rates may be influenced by an individual’s genetic background (Conrad et al., 2011). The notion that genetic variants in the genetic background which are capable of significantly influencing the penetrance of disease was also previously suggested (Demogines et al., 2008). Indeed, investigations have shown that patients with schizophrenia caused by new mutations, displayed generally increased mutation rates compared to control individuals (Girard et al., 2011; Xu et al., 2012).

### Locus Heterogeneity

The pleotropic effect refers to the situation where a single locus affects more than one phenotypic trait, and plays established roles in the manifestation of genetic disease (Stearns, 2010). In contrast, locus heterogeneity describes the situation where a particular phenotypic trait or disorder can result from mutations at any one of several distinct loci; this particular concept likely plays key roles within the proposed framework, as it could potentially help explain the more frequent occurrence of complex genetic diseases within the population. A proposal of locus heterogeneity would also have equally important implications for other observations regarding the manifestation characteristics of some of these diseases. For example, it is sometimes observed that some families affected by a particular multifactorial disease have a higher incidence risk than others, most often amongst siblings. In fact if a couple have two children with a particular disease, the recurrence risk for the next child steeply increases. Within these families, it is possible that the particular causative gene is an example of a mutation hotspot locus thus significantly increasing the risk to offspring. Additionally, the parents could harbor a high number of mutator alleles/low number of anti-mutator alleles, thus further increasing the risk. Another observation in a few examples of multifactorial disease, such as cleft palate and cleft lip and neural tube defects, is that if a child is severely affected with the disease, then the recurrence-risk for next child also increases. This is a little more difficult to fully explain with the current framework, however, the proposed locus heterogeneity likely also plays a key role: some genes will encode factors which when disrupted cause different aspects of a biological process or physiological system to fail, thus though they manifest as the same observable disease, they do so with varying degrees of severity.

## Considering Characteristics Of Disease Manifestation And Sex Dimorphism

Many multifactorial diseases display a dichotomous manifestation. This is most apparent in the congenital diseases such as cleft palate and cleft lip, pyloric stenosis, clubfoot, and neural tube defects, but also in others such as autism, schizophrenia, and bipolar disorder. These and others are much better explained by the current single-gene model than by the polygenic model, as the former does not require invoking the idea of disease-thresholds, the actual biological mechanisms for which are ill-defined.

As described earlier, the Carter effect is posited as an explanation for observations of sex dimorphism in the polygenic threshold model, for which no actual proposed biological mechanism exists. Within the current framework I suggest that the examples of sex dimorphic multifactorial diseases might instead be explained by X-linked inheritance, which only occurs in some families due to the high degree of locus heterogeneity. For diseases which display a rather high level of sex dimorphism such as in pyloric stenosis, clubfoot, and autism, more of the causative genes or even just one or two which are mutation hotspots could be located on the X chromosome; whereas those that display a more modest degree of dimorphism contain fewer, perhaps even just one causative X-chromosome gene. When X-linked inheritance does occur within families, somatic mosaicism complicates the pattern of X-linked inheritance expected from the classic Mendelian prediction much more frequently than it normally would do. For example, since random X-inactivation occurs at the ∼20 cell stage of the developing female embryo, and since somatic mosaicism is inherent in determining disease occurrence under this framework (i.e., only a smaller amount of tissue needs to be affected), then it is much more likely that random X-inactivation could lead to manifestation of the disease, as inactivation of the normal X-chromosome would only need to occur in a much more limited amount of tissue. Thus in such situations, daughters of mother-carriers would be more frequently affected than expected, thus masking the pattern of X-linked inheritance that we expect from the usual Mendelian prediction where males are mainly affected after inheriting the faulty gene from a carrier mother.

Indeed, the most common cases of sex dimorphism in multifactorial disease are where the number of affected males significantly outnumber the number of affected females within the population. In such cases, if the Carter effect does occur, the most readily detectable observation would be that relatives of affected females should be at higher risk than relatives of affected males. A Danish study which included a cohort of ∼two million children, ∼3,400 of which had surgery for pyloric stenosis, found no such correlation (Krogh et al., 2010). In a second further large-scale study, it was reported that the recurrence rates of autism spectrum disorders (ASDs) were the same in families with affected female individuals as with affected male individuals (Ozonoff et al., 2011). Indeed, the authors of both of these large-scale studies commented that the observed data do not support the polygenic threshold model. An interesting observation, however, was that maternal half-siblings are at a significantly increased risk compared to paternal half-siblings in a large Danish study reporting on ASD incidence (Grønborg et al., 2013). The authors propose, that factors associated with pregnancy and the maternal intrauterine environment contribute to the risk of developing ASDs; but, the observations may instead be explained if females are more often carriers of a faulty gene(s), i.e., one that is X-linked. As is apparent, only a very limited amount of information can be gleaned from such studies, and the complications to predicted patterns of inheritance added by the current framework exacerbates these limitations.

## Other Important Considerations

### Genetic Interactions

In Fisher’s (1918) framework, genetic factors contribute to a continuous trait, such as height, in an additive fashion. It’s a revered theory amongst geneticists and describes continuous traits, in an elegant, easy to envisage manner. However, when used in this fashion in attempts to account for common diseases within populations, shortcomings become apparent (Nelson et al., 2013); and multiplicative models have been suggested where gene–gene interactions likely play a significant role (Risch, 1990; Wray and Goddard, 2010). Higher order genetic interactions (HGIs) probably do exist in nature (Taylor and Ehrenreich, 2015), though the roles for these in disease manifestation have not previously been described, and we have knowledge of only very few examples of them in biological systems. It has been suggested that genetic studies fail to detect HGIs, because of a lack of statistical power (Cordell, 2009; Taylor and Ehrenreich, 2015), but even so, it may indeed be that the roles in disease manifestation are actually rare. This is since when considering multiplicative genetic interactions from a biological perspective, we are referring to specific genetically encoded allelic products, rather than any form of the genetically encoded product, that are able to influence each other in a biologically meaningful way. Although attractive as a concept in genetics, from a cell biology point of view, this is very difficult to imagine as occurring with any meaningful frequency, as would be expected in the polygenic model. Genetic background, however, is a well-recognized biological feature in influencing phenotypic variation (Nadeau, 2001; Dowell et al., 2010; Taylor and Ehrenreich, 2015). In the current framework though mutator/anti-mutator variants are introduced into the genetic background, they most likely act in an additive fashion, as described by Fisher (1918), to influence an individual’s general mutation rate.

### Recessiveness vs. Dominance

As recently discussed by Henn et al., 2015, dominance is perhaps the most important quantity that has not been estimated from genome-wide data characterizing the proportion of deleterious mutations within genomes, though it’s fair to stipulate that the proportion of deleterious mutations that are recessive or dominant remains an open question. However, we know particularly from examples of classic Mendelian disease but even some cases of sporadic genetic disease, that a recessive mode is the most commonly observed route to disease manifestation. Thus for a particular locus, examples where half the amount of ‘wild-type’ genetic product is insufficient, or where a variant product is dominant over a ‘wild-type’ product, are in fact rare. Though this is what the polygenic disease model generally assumes, and it assumes that this occurs with a high degree of frequency, particularly in the rare-variant hypothesis where new genetic variants are almost exclusively found in heterozygous form. The two-hit model presented in the current framework instead posits a generally recessive mode of inheritance where both copies of a gene need to be inactivated.

### Contributions from the Environment

Multifactorial disease are always thought of as having an environmental component that also significantly contributes to disease risk. Sometimes it is much more obvious what the environmental components may be; they include diet, exercise, and smoking habits for conditions such as hypertension, diabetes, obesity, and cancer for example. In other cases the factors are much more difficult to ascertain, and this is particularly the case for multifactorial congenital disorders. It has been shown for example that the concordance rates for monozygotic twins in clubfoot, cleft lip, and pyloric stenosis is fairly modest, ranging from 17 to 46% (Christensen and Fogh-Anderson, 1993; Engell et al., 2006; Krogh et al., 2010). Pyloric stenosis is not truly congenital, in that it becomes apparent in the first 2–8 weeks of life; and based on previous suggestions, Krogh et al. (2010) attribute the 54% discordance amongst monozygotic twins from their large-scale study to factors such as feeding practice, maternal smoking, infant sleeping position, and postnatal use of macrolides, as possible contributing environmental factors. For clubfoot and cleft lip and palate, environmental factors that have been suggested are maternal smoking and alcohol intake. Although some of these environmental factors are sometimes responsible for the incidence of sporadic birth defects, their contributions to the aforementioned multifactorial diseases, and others, may have been overestimated. Instead, discordance between monozygotic twins may be much more readily explained by the current two-hit framework, where the second post-zygotic hit is required for disease manifestation.

## Testing The Model And Demonstrating Genotype–Phenotype Causality

Currently, much focus in identifying associative genetic factors in complex genetic disease is on RVAS studies, and it has been suggested that such population-based studies need to be of a substantially large sample size in order to be able to detect the causal rare variants (Lee et al., 2014; Robinson et al., 2014; Sham and Purcell, 2014). The appropriateness of this line of investigation can be questioned (Wagenmakers et al., 2014). It has also been suggested that smaller family-based studies would be ineffective in disease-gene identification (Robinson et al., 2014), though this observation is based on a framework where an underlying polygenic architecture exists. Although in the framework proposed here, the disease-causing alleles are likely to be rare variants, family-based studies rather than large-scale population based studies would be better suited for disease-gene identification. The framework posits a single causative gene, however, segregation-analysis would not be entirely appropriate, as unaffected siblings may also harbor the inherited mutation. Instead blood/cheek swabs from parents and affected individuals should be obtained for sequencing, but also critical here is the identification of the second hit in affected tissues from the affected individuals; indeed testing of unaffected vs. affected tissues was previously used to identify causative mutations in sporadic forms of Proteus syndrome (Lindhurst et al., 2011). It may not always be possible to obtain affected tissue from living patients, but wherever it is feasible to obtain such material in a stress-free manner, they should be biopsied and sequenced. In some situations identifying the precise regions of tissues that are affected may be less-obvious, in which case tissue of the appropriate developmental lineage may instead be analyzed. In other cases, a mixture of normal/affected tissue might be unavoidable, however, next-generation-sequencing approaches and recent developments in computational methods should be able to identify and distinguish the somatic mutations (Roth et al., 2012; Schmitt et al., 2012; Kim et al., 2013; Josephidou et al., 2015). A key consideration is that it would need to be determined that the second hit has occurred on the opposite chromosome and not in the same copy of the gene that harbors the first hit. This may now be possible with Nanopore sequencing methods which allows the direct DNA sequencing of ultra-long reads, which would allow haplotypes to be readily determined. Although not yet commercially available, Oxford Nanopore Technologies (ONT), have made available their first sequencing device (the minION) as part of an early-access program for researchers. Early reports have indeed confirmed the ability of the device to obtain sequencing reads of tens of kilobases in length (Laver et al., 2015). Once biallelic hits in a gene from the affected tissue have been identified, only one of which would be more likely be present in unaffected tissue, and they are both predicted to significantly disrupt gene function, they should be considered candidate genes. However, demonstration of causality needs to be a central consideration, and should be assessed by biological means. Animal models may be useful in such regards, but care must be taken to recapitulate as closely as possible the physiological manifestation characteristics of the disease, i.e., the second hit needs to be induced in the correct developmental lineage and in a relevant point in time, which may not always be readily identifiable. However, cheaper and quicker genome editing methods, such as the Crispr-Cas9 system, mean that such investigation can be more routinely performed, and such biological tools would also further be useful in defining and characterizing routes to therapy.

Depending on the degree of locus heterogeneity, several disease genes may exist for a particular disease, one of which will be disrupted in each family. Each of these genes will first need to be identified as described above, but thereafter, blood/cheek swab mutation screening of these candidate genes could be performed in all families for future diagnostics applications. Research costs are often a limiting factor in medical genetics, but it is important that all of the causative disease genes for a particular disease are comprehensively identified, if at least to provide a more satisfactory level of genetic counseling than is currently available for all families affected. In this regard, yet another very alluring feature of ONT devices is their low cost, since although the sequencing cost per genome is rapidly improving for the currently commercially available technologies (van Dijk et al., 2014; Watson, 2014), these generally operate via a limited number of core sequencing facilities. However, the ONT devices themselves will be much more routinely affordable and we may thus envisage smaller family-based studies which all or most medical genetics laboratories around the world are able to perform; this should speed up comprehensive disease gene-identification, providing much better defined routes to therapy in a family-tailored manner. Roughly 85% of disease causing mutations identified in classic Mendelian disease occurs in the protein-coding portions of the genome, and it is probably likely we would see a similar percentage under the current framework. Therefore, where cost is an especially limiting factor, studies might focus on whole-exome-sequencing approaches, until whole-genome-sequencing becomes more routinely affordable. Finally, since an advantage of this framework is that it explains diseases that have a more dichotomous manifestation better than the polygenic model does, these particular diseases may be more suitable for initial investigations.

## Concluding Remarks

The primary, perhaps only reason we should harbor hopes that such a framework is correct, is that it would make it considerably easier to define and develop routes to therapy, as this could be specifically targeted against the single causative monogenic factor within families. Such hopes of course have little or nothing to do with scientific reality and it is very possible that the framework presented here is incorrect. The jury is still very much out for the polygenic rare-variant hypothesis, and the previous overwhelming consensus regarding the polygenic common-variant hypothesis was, for most intents and purposes, wrong. I thus feel rather apprehensive to conclude with a comment regarding how likely it is for this proposed framework to hold true to fact. However, the framework appears broadly consistent with recorded observations past and present, and if correct, the potential benefits are huge; testing of the model is clearly incumbent.

## Conflict of Interest Statement

The author declares that the research was conducted in the absence of any commercial or financial relationships that could be construed as a potential conflict of interest.
